# Dental Caries among Refugees in Europe: A Systematic Literature Review

**DOI:** 10.3390/ijerph17249510

**Published:** 2020-12-18

**Authors:** Sneha Bhusari, Chiamaka Ilechukwu, Abdelrahman Elwishahy, Olaf Horstick, Volker Winkler, Khatia Antia

**Affiliations:** Heidelberg Institute of Global Health, Heidelberg University Hospital, 69120 Heidelberg, Germany; sneha.bhusari@uni-heidelberg.de (S.B.); nkorika.ilechukwu@alumni.uni-heidelberg.de (C.I.); abdelrhman.elwishahy@alumni.uni-heidelberg.de (A.E.); olaf.horstick@uni-heidelberg.de (O.H.); volker.winkler@uni-heidelberg.de (V.W.)

**Keywords:** caries, decay, Decayed Missing and Filled index (DMF) and dental health, refugee, asylum seeker

## Abstract

Oral health is one of the most neglected aspects of refugee health. The study aimed to systematically review evidence on prevalence of dental caries and dental care services provided to refugees in Europe. Following PRISMA guidelines, we searched PubMed, Cochrane, WHOLIS, Web of Science, Medline Ovid, and Google Scholar identifying studies on dental caries among refugees in Europe after the 2015 refugee crisis. From 3160 records, fourteen studies were included in the analysis. Eight studies on oral health showed caries prevalence of between 50% and 100%, while it ranged from 3% to 65% in six general health studies. Caries prevalence was proportional to age and inversely associated with education, whereas gender and country of origin showed no significant association. Nowhere is oral health part of general health assessment on arrival and is complaint based. Primary focus on resettlement, language, cultural, and economic barriers emerged as explanatory models for limited access. Our study identified a high prevalence of caries and limited access to dental health services as main challenges. Integrating oral health check-ups may contribute in shifting towards preventive oral care. Further research is urgently needed to better understand the dental needs of refugees in Europe.

## 1. Introduction

Antonio Guterres, the Secretary-General of the United Nations, described the European situation in 2015 as “primarily a refugee crisis, not only a migration phenomenon” [1]. In 2015, the largest movement of people for 20 years was seen, with more than 3.5 million refugees in Europe [2]. The International Organization for Migration (IOM) defines a refugee as “a person who is outside the country of his nationality and is unable to avail himself of the protection of that country” [3]. During refugees’ state of unrest, the most valuable assets become necessities such as clean water, food, nutrition, shelter, sanitation, and protection, while medical assessment and healthcare are neglected [4]. Moreover, refugees are faced with language barriers, unfamiliar surroundings, new laws, rules and regulations [3].

Refugees are always at a risk for innumerable issues regarding health [5]. Of all the conditions faced, necessary or emergency health issues are addressed in European countries [6]. Major areas of health focused upon include non-communicable and communicable diseases, maternal and child health, occupational health and mental health [7]. Even though oral health is a key indicator of overall health, well-being and quality of life [8], it is not part of this essential list [7]. Moreover, the exclusion of dental assessment within basic care makes refugees more vulnerable [4] and the lack of active involvement of a dentist curtails the importance of oral health [9].

Oral and or dental diseases are correlated with non-communicable diseases (NCDs) [9]. They can result in malnutrition due to alterations in diet, and phonation problems, especially in the older age group [10]. There is also higher body dissatisfaction [11] and simple acts of smiling, communicating and eating can be affected negatively [12]. Hence, oral health not only affects one’s general health, but also has an impact on mental health. Dental caries is the leading oral health problem, with high prevalence, affecting a large population in the majority of the countries, including Europe [12,13]. The basic motive for seeking oral health is mainly pain based [12]. Ordinarily, oral health, and in particularly caries, is one of the most neglected aspects of health irrespective of region, culture, education or the socioeconomic status of an individual, and more so in low and middle-income countries. The overall burden is decreasing due to public health measures, but prevalence still remains high [12].

Considering war-affected regions, attention to oral health can be even worse or non-existent. Such populations suffer the most, not only with the general requirement for oral care, but also with need based (i.e., pain based) oral care, and prioritizing oral health becomes increasingly difficult for refugees as other priorities are pre-eminent [6]. In light of this situation, the prevalence of dental caries is expected to be high among refugees in general and in Europe in particular. Lack of proper education, information and awareness of oral health, lack of inclination to maintain good oral health, overall neglect of oral health and financial limitations, coupled with geographical constraints, war or devastating surroundings, migration, resettlement in foreign lands, language barriers and lack of stability have resulted in an increase in dental caries (along with other oral problems) [14]. This lack of provision is the main area of concern about, and hindrance to, obtaining health data and achieving good health care.

The aim of this study was to find out the prevalence of dental caries among refugees in the European region. The objectives were twofold: first, to synthesize the evidence of prevalence of dental caries among refugees in the European region after the 2015 crisis by evaluating the Decayed Missing and Filled index (DMF); and second, to evaluate the dental care services provided to the refugees in Europe and their needs and shortcomings

## 2. Materials and Methods

This study followed the reporting guidelines of Preferred Reporting Items for Systematic Reviews and Meta-Analyses (PRISMA) [15]. We included all types of quantitative and qualitative study. There was no restriction regarding the language, age, gender, country of origin, education, or socioeconomic status of the participants. However, we included only studies focusing on caries among refugees or asylum seekers after 2015 (the European Migrant Crisis), but not on oral conditions of periodontium or oral mucosa. We also excluded studies with the word “migration”, “migrant” or “immigrant” from the search. The word ‘Europe’ was a broad term; therefore, we dropped the term ‘Europe’ during the database search and manually searched for European studies. We performed the search in English using the key words Refugee or Asylum seeker in combination with Caries, Decay, DMF or Dental Health through the following databases: PubMed, Cochrane, World Health Organization Library Information System (WHOLIS), Web of Science, Medline Ovid and Google Scholar. The search was finalized on 21st November 2020. The database searches as well as the screening procedure were run independently by the first and the second author. Conflicts were resolved upon agreement by focusing on the eligibility criteria and the aims set for this review. Removal of duplicates was carried out at a later stage. Table 1 denotes the PICO criteria used for this study.

The decayed, missed and filled index, known as the DMF index, is the measure of the prevalence of caries; it identifies the number of teeth with dental caries including its effects on an individual [16]. The DMF index has been a simple, rapid, universally accepted and widely used tool for several decades to determine coronal caries experience, since it requires a minimal inventory: natural light, plain mouth mirror and a fine probe. The calculation of DMF is performed by obtaining the number of decayed, missed and filled teeth or surfaces [17,18]. However, the DMF index does not distinguish the reason for loss of tooth (MT) [16]. We extracted information regarding Decayed teeth (DT), Missing teeth (MT), and Filled teeth (FT), and the average DMF index from the included studies to look mainly at the experience of caries, where only the DT factor was focused on.

We evaluated risk of bias using the quality assessment tool of The U.S. National Institute of Health (NIH) [19]. This provided separate assessment criteria for different types of studies under one domain. We used two groups: Quality Assessment Tool for Observational Cohort and Cross-Sectional Studies, and Quality Assessment Tool for Case Series Studies. 

## 3. Results

**Figure 1:**
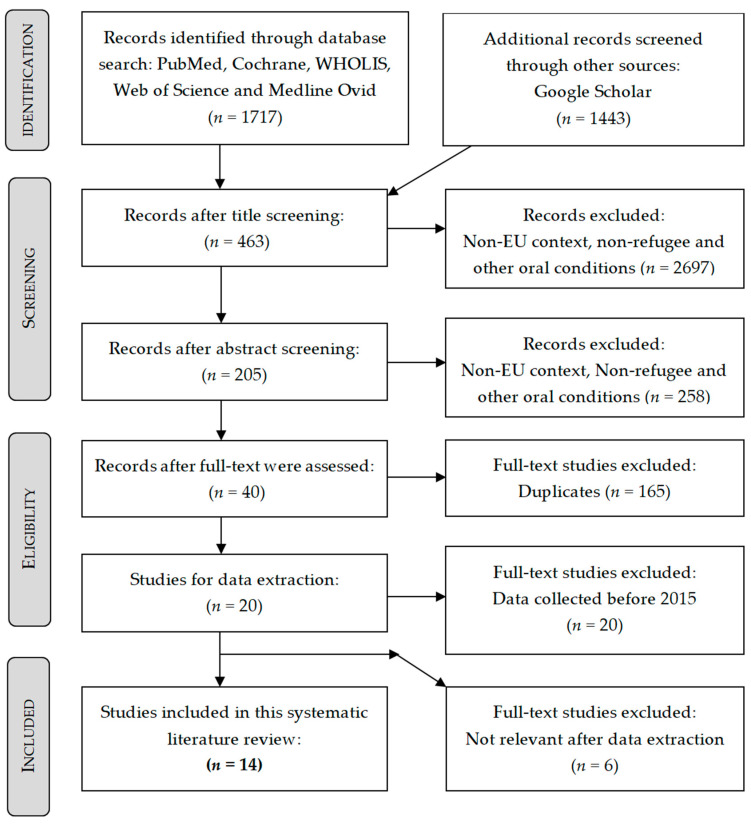
Preferred Reporting Items for Systematic Reviews and Meta-Analyses PRISMA flow chart depicting the selection process [15].

Our search yielded 3160 records, 1717 from the five databases and 1443 from Google scholar for which we screened the first 200 hits per combination until no further relevant studies were found. 205 articles remained after title and abstract screening, from which twenty full texts were evaluated against the eligibility criteria. Finally, fourteen studies were included in this systematic literature review. A detailed description of the screening process can be seen in Figure 1.

From the final fourteen articles, only one was a qualitative study [20] while others were: ten cross-sectional [6,21,22,23,24,25,26,27,28,29], one cohort study [30] and two case reports [2,13]. All except two studies [13,28] had a comparison group. By the Quality Assessment Tool, only one was graded as ‘fair’ [30] while all other studies were ‘good’. The main host countries were Belgium, Finland, Germany, Greece, Norway, Spain, Sweden and the UK, while refugees originated from a wide range of countries with a majority coming from Afghanistan, Iraq and Syria. Less frequently, refugees came from Asia, Africa, Europe and the Middle East as listed in Table 2.

All studies addressed information on oral or health status, four studies [6,13,29,30] focused on the healthcare needed, three studies [20,25,28] examined the treatment provided and two studies [13,30] examined necessary improvements. The study populations in three studies [24,25,29] were children, in five studies children and adults [22,26,27,28,30] and in four studies only adults [6,20,21,23]. All study samples consisted of more men than women. Questionnaires on both medical and travel history along with present living and medical conditions including clinical assessments were the primary sources of data in all studies except one [20], which used self-reporting as a source.

Only eight studies [6,13,20,21,23,24,27,28] had oral health as the focus while others concentrated on oral checkups along with general health assessments. The prevalence of caries was higher in the oral health focused studies as shown in Table 3. Only one study [24] reported a low prevalence of caries, which was explained by the fact that children from wealthier families had better access to oral health services in the country of origin. From the above mentioned eight studies, only five studies [6,21,23,24,27] used the DMF index as a part of their analysis, while others recorded oral issues based on complaint. Four studies [6,21,23,27] out of the five reported a very high DMF severity. These five studies also showed an expanded version of the Decayed Missing Filled Teeth (DMFT) index with individual components reported, namely Decayed Teeth (DT), Missing Teeth (MT) and Filled Teeth (FT). As seen in Table 4, Table 5 and Table 6, the DT is observed to be high for all the studies except one [23], where the author suggests the high to moderate social status of the sample population to be the reason for higher MT and FT as compared with DT. DMF is denoted as an index for permanent teeth, and dmf for deciduous teeth.

Six studies found an association between caries and socio-demographic variables [6,21,22,24,27,30]. Age was directly correlated while education was inversely proportional to caries prevalence [30]. Caries was inversely proportional in deciduous dentition age while directly proportional to permanent dentition age [24]. All studies showed that men had a higher prevalence compared to women. No country of origin specific effects were observed [22] but Høyvik et al. [6] suggest that the differences in caries prevalence are related to the origin of the refugee population when comparing two sets of refugees from the Middle East and Africa. None of the included studies had access to the pre-arrival oral health status of the sample population.

All except four studies [13,20,22,24] showed the need for oral screening and all except five studies [2,6,22,25,30] concluded the need for a preventive focus. Freiberg et al. [28] suggested that regular check-ups have a potential to improve refugees’ health literacy and raise awareness of the benefits of such preventive measures. The utilization of an existing Primary Health Center (PHC) to incorporate oral health care need was suggested in six studies [20,21,22,25,26,30]. Furthermore, seven studies [6,20,21,22,23,25,26] pointed to the economic burden on both the refugees and on the host country while dealing with easily preventable oral complications. General referral systems seemed to be in place according to four studies [23,25,26,30] while two studies [13,20] directly provided necessary interventions. Specifics about utility of referral systems were not discussed in any of these studies. Six studies [6,21,24,27,28,29] emphasized the need for interventions. Moreover, Al-Ani et al. [27] encouraged all European migrant receiving countries to strengthen their dental capacity, as refugees’ dental care needs are expected to further increase in the near future. Accessibility, cariogenic diet and poor oral hygiene were seen to be the main causes for disease pattern in all the included studies. The study of Hjern and Kling [29] argued that children are especially vulnerable, as they are affected by the caries-promoting food culture of their families. Finally, five studies [2,20,21,23,27] raised the issue of ‘Health as a human right’. One [2] study stressed the importance of clinicians to carry out a dual role by providing care and advocating for dental needs. Language and cultural barriers [2,6,13,20,21,25,27,28], selection bias, mainly due to self-reporting or voluntary treatment-seeking behavior, among other reasons [6,20,21,22,23,26], lack of diagnostic tools and resources [6,13,21,22,26], small sample size [20,23,24], missed other oral health details [6,21,22,23,24,26], lack of representativeness [21,25,28] generalizability [22,25], crude methods used and insufficient data quality [27] were some of the limitations reported in the included studies.

### Further Results

Our study found that refugees are at increased risk of developing oral diseases (mainly dental caries) when compared to the local populations (See Table 3). Filled Teeth were more frequent among the local populations and also among other migrants in comparison to refugees, whereas Decayed Teeth were more common among refugees (See Table 3). This clearly shows that the local population has better access to and utilization of available dental treatment. Missing Teeth were similarly distributed among all three groups (See Table 3). The authors explained this by the fact that refugees originated mainly from war-affected regions, where the priority for curative treatment is completely absent [24]. Availability of health services seems to be scarce, along with other necessities such as clean drinking water, a hygienic environment and other cleaning and sanitation products [23]. Moreover, children tend to suffer more since they are not provided with the essential oral health services and practices, which may have long-lasting negative effects [23,29]. Our findings show the need for oral health assessment tools such as overhead light, mouth mirror, probe/explorer and intra oral *x*-ray/orthopantomogram to aptly collect the data [6,13,21,22,26]. The studies emphasized the lack of human and material resources [6,20,23,25]. A shift from curative to conservative to preventive care is highly recommended [13,20,21,23,24,26,27,28,29].

Effects of oral health on refugees’ general health is an important aspect addressed by several studies; e.g., Høyvik et al. [6] state that dental problems have a substantial effect on social, physical and psychological well-being; missing teeth can be detrimental to self-confidence. Especially, reduced social and psychological well-being can delay the acceptance and amalgamation process and, therefore, lead to social isolation and mental issues resulting in increased overall health problems [23]. Other factors not directly associated, but important, such as dental fear, anxiety or post-traumatic stress disorder (PTSD), also need appropriate planning and time for treatment [2,6,13,20,26,30]. Additionally, the unavailability of orientation from the host country [21] and of proper oral care is one aspect highlighted by all studies except one [30].

Studies included in our analysis emphasize the health needs and oral health seeking behavior of refugees. Findings suggest less motivation and orientation regarding oral health care and prevention among refugees when compared to the local population [21]. Refugees’ priorities tend to be more towards resettlement [6]. Additionally, studies suggest that refugees in the transition phase are provided mainly with emergency care. Refugees tend to have similar access to dental services as the local population only once their refugee status is accepted [2,20,21,25,26,28]. Language, cultural and economic barriers, social isolation, the unfamiliarity of the health care system of the host country, laws, regulations and restrictions can further limit access to needed dental care.

## 4. Discussion

Prevalence of caries and dental treatment needs are high among refugees and the burden is increasing with the ever-growing influx of this population. The complex process of integration entails challenges, which also puts a burden on the host country. The unavailability of oral screening at reception sites leads to missing detectable oral health problems, which should be treated as early as possible to improve treatability. Consistent with our results, other studies from the USA [31,32,33], Canada [34] and from Australia [35,36] show high prevalence of caries, poor oral hygiene and similar unmet treatment needs among the refugee population. Moreover, a lack of information on pre-arrival oral status makes comparison and assessment difficult.

We found that more data is available on the general health needs of refugees in Europe while data on oral health is scarce. Additionally, the lack of oral assessments and inconsistencies within insurance systems, such as lack of uniformity and harmonization in cost coverage which depends on per capita spending on health care [37], add to the barriers in achieving good oral health. As a result of these large fluctuations and the diversity of refugees, the challenges faced are not homogenous [6]. The journey, and later the waiting time to become an officially accepted refugee by the host country, exacerbate preexisting conditions [21]. This not only increases suffering, but also incurs unnecessary costs [6]. This, in turn, puts excessive pressure on the individual as well as the host country’s health system [21]. Language, cultural barriers and the unfamiliarity of the health system further amplify this. The European refugee crisis is a persistent issue gripping refugees and host countries alike and brings in challenges on a daily basis. In spite of all the advancements and resources available at the disposal of European countries with a good health care system in place, inclusion and integration of refugees and asylum seekers still remains challenging [6,7,23].

Studies that primarily focused on refugee oral health examined oral hygiene practices, periodontal health, DMF of teeth and knowledge and self-perception regarding oral health. A dentist and necessary dental equipment were also available for the assessment, making it easier to detect problems. However, equipment for screening, such as dental *x*-rays, was not available which might have led to an underestimation of prevalence. Studies that focused on general health had no dentists in their study teams and dental equipment was not mentioned. Hence, only complaints about oral/teeth problems or pain were registered, which is likely to have led to an underestimated prevalence of oral problems.

Studies included in this review clearly show the substantial effect of oral health on general health and especially on mental health and well-being. Some non-migration focused studies investigated the link between oral and general health, e.g., Kitamoto et al. [38] and Patini [39] suggest an association between oral microbiota and systemic diseases.

Dental fear and anxiety are other important aspects emphasized by the included studies. Especially, children seem to be more vulnerable to pain associated with dental treatment. Some authors examine this issue and emphasize the importance of local anesthesia (LA) in achieving pain-free treatment [40]. However, according to the authors, anxiety and stress associated with local anesthetic injection makes pain-free treatment challenging [41,42].

Little emphasis has been given to oral health research among refugees in European countries during the last 25 years [6]. Further research is needed; however, based on available data, targeted interventions should be implemented [6]. Early detection of oral health conditions can be considered as the most effective way to address the complex problem of oral health. Immediate oral assessment of refugees at the point of entry or registration for consecutive dental screening [21] can prove vital [6]. Communication in the native language can also help avoid any misunderstanding and delays [21].

Consistent with our results, studies from other non-European regions suggest that targeted services will help access major oral health care challenges even with limited resources [6]. Riatto et al. [24] state that a structured assessment of the refugees’ situation with respect to the amount of dental care received, economic capability, knowledge and awareness, and access to oral health care services will be needed to plan and arrange necessary services for oral health care. Canada is the only country with specific guidelines for oral services for refugees [43].

Several limitations of this systematic review should be mentioned. No language restrictions were set during the search but the search terms were in English. Due to its simplicity and popularity, we focused on the DMF index as a quantitative measure of caries prevalence. We only concentrated on the D factor of the DMF index. As MT can also be due to multiple reasons other than caries (such as trauma, periodontal issues, etc.), there can be a risk of bias. However, our search and screening procedure did not bring up other measures to quantify caries among refugees; only one study used Index of Restoration (IR) [24]. Due to the lack of comparability, we decided not to perform a formal meta-analysis. Due to unavailability of data, we could not compare pre and post-arrival oral health conditions. Lastly, the element of human error and bias cannot be neglected, which may have caused the loss of some information or a steering of the conclusions.

Despite these limitations, our study provides a comprehensive analysis of the available data on dental caries and provided oral health care among the refugee population in the European region after 2015. This systematic literature review adds to the existing literature on the specific needs and associations required for further planning. Moreover, it brings dental and oral health into focus. Concentrating on caries may help to discretely tackle a major condition, provide required treatment and precisely fulfill unmet needs for better oral health.

## 5. Conclusions

Our systematic literature review shows a high burden of dental caries, with increasing severity among refugees in Europe. Factors such as pre-existing poor oral health, limited access to treatment, language and cultural barriers, and lack of orientation and unfamiliarity with the host country’s health care system might be major reasons and lead to low oral health-seeking behavior. Additionally, dietary behavior and changes owing to migration, low oral hygiene practices and lack of preventive measures in the host countries lead to worse oral health over time. Further research focusing on refugees in Europe is needed to better understand, plan and install preventive measures. Setting priorities now with the available data is urgently needed to improve oral health among refugees in Europe.

### Key Points

(1) High prevalence of caries and limited access to dental health services are the main challenges refugees and asylum seekers face in Europe.
(2) Further research is urgently needed to better understand the dental health needs of refugees in Europe.
(3) The necessity of oral health check-up irrespective of need will help make the shift from curative to preventive oral health care.

## Figures and Tables

**Table 1:** PICO and eligibility criteria for this systematic literature review.

Criteria	Inclusion	Exclusion
Population	Refugee and Asylum seeker	Migration, migrant, immigrant
Indicator	European region	Other regions
Comparison	No specific comparators set	No specific comparators set
Outcome	Caries, Decay, DMF, Dental health	Other oral conditions of periodontium or oral mucosa

**Table 2:** Characteristics of Included Studies by hierarchy of evidence.

Author	Year	Study Type	Sample Size	Age in Years	Host Country	Country of Origin
[30] Hermans et al.	2017	Dynamic cohort	2291	18–38	Greece	Afghanistan, Pakistan and Syria
[21] Solyman and Schmidt-Westhausen	2018	Cross-sectional	386	18–60	Germany	Iraq and Syria
[22] Kakalou et al.	2018	Cross-sectional: Descriptive	6688	0–75+	Greece	Afghanistan, Iraq and Syria, other regions: Africa, Asia and the Middle East
[6] Høyvik et al.	2019	Cross-sectional: Comparative	132	18–47	Norway	Africa and The Middle East
[23] Goetz et al.	2018	Cross-sectional: Pilot	102	16–64	Germany	Afghanistan, Armenia, Chechnya, Eritrea, Iran, Iraq, Somalia, Syria and Yemen
[24] Riatto et al.	2018	Cross-sectional	156	5–13	Spain	Syria
[25] Pavlopoulou et al.	2017	Cross-sectional: Prospective	300	0–14	Greece	Afghanistan, Bangladesh, DR Congo, Eritrea, Iran, Kenya, Lebanon, Pakistan, Somalia and Sudan
[26] van Berlaer et al.	2016	Cross-sectional: Descriptive	3907	0–75+	Belgium	Afghanistan Iraq, Morocco, Palestine and Syria
[20] Mattila et al.	2016	Cross-sectional: Pilot	38	17–53	Finland	Afghanistan, Hungary, Iran, Iraq, Morocco, Russia, Slovakia, China, Somalia, South Sudan, Sweden, Syria, Thailand, Turkey and Vietnam
[27] Al-Ani et al.	2020	Cross-sectional	544	3–75+	Germany	Mainly from Afghanistan, Iraq and Syria, Others nationalities: African countries, Arabian countries, Asia and Eastern Europe
[29] Hjern and Kling	2019	Cross-sectional	639	6–15	Sweden	Afghanistan and Syria
[28] Freiberg et al.	2020	Retrospective observational	568	20–34	Germany	Afghanistan, Iran, Somalia and Syria
[13] Zaheer et al.	2017	Case report	NS	NS	Greece	Afghanistan, Kurdistan, Iraq and Syria
[2] Williams et al.	2016	Case report	NS	NS	European mainland and the UK	Afghanistan, Albania, Eritrea, Iran, Iraq and Syria

Note: NS—Not specified.

**Table 3:** DMF and Caries prevalence.

Study	Focus	Dentist Involved	Instruments Mentioned	DMF	Prevalence of Caries %	Reliability Tested	Guideline
[30] Hermans et al.	GH	NR	NR	NR	2.9 *	NR	SPHERE
[21] Solyman and Schmidt-Westhausen	OH	Yes	Yes	Yes ^a^	87.5	Yes	WHO
[22] Kakalou et al.	GH	NR	NR	NR	4.6	NR	ICD-10
[6] Høyvik et al.	OH	Yes	Yes	Yes ^a^	89.4	Yes	astdd
[23] Goetz et al.	OH	Yes	Yes	Yes ^a^	NR	1 Dentist	ICDAS (STROBE)
[24] Riatto et al.	OH	Yes	Yes	Yes ^b^	(50–75)	Yes	WHO
[25] Pavlopoulou et al.	GH	NR	NR	NR	24.7	NR	NR
[26] van Berlaer et al.	GH	NR	NR	NR	8.1	NR	ICD-10
[20] Mattila et al.	OH	Yes	NR	NR	AS 57	NR	NR
[27] Al-Ani et al.	OH	Yes	Yes	Yes ^c^	Age groups:	Yes	WHO
					0–3 (49)		
					6–11 (14)		
					13–17 (28)		
					18–34 (10)		
					35–44 (16)		
					45–64 (21)		
[29] Hjern and Kling	GH	NR	Yes	NR	48.1	NR	NR
[28] Freiberg et al.	OH	Yes	NR	NR	98.7	NR	(BEMA)
[13] Zaheer et al.	OH	Yes	NR	NR	100	NR	NR
[2] Williams et al.	GH	NR	NR	NR	65	NR	NR

Note: GH—General health; OH—Oral health; AS—Asylum seeker; NR—Not Reported; * For 30 Patients; ^a^ All participants were adults; ^b^ All participants were children; ^c^ Participants were children and adults. For deciduous (up to 6 years—permanent (up to 12 years) teeth respectively. SPHERE—Global movement started in 1997 to improve quality of humanitarian assistance, Humanitarian Charter and Minimum Standards in Humanitarian Response; WHO—World health organization; ICD-10—International Classification of Diseases, 10th revision; astdd—Association of State and Territorial Dental Directors; ICDAS—International Caries Detection and Assessment System; BEMA—standard of evaluation of dental services and forms within the statutory health insurance in Germany.

**Table 4:** Detailed DMF Index reported in the included (dental) studies: All participants were adults.

Study	Average/Mean							
	DMFT		DT		MT		FT	
[21] Solyman and Schmidt-Westhausen, 2018	6.4		4.0		1.5		0.9	
[6] Høyvik et al., 2019	7.4		4.3		1.4		1.7	
	ME	A	ME	A	ME	A	ME	A
	10.7	5.7	5.2	3.9	1.6	1.3	3.9	0.5
[23] Goetz et al., 2018	6.9		2.9		3.9		3.8	

Note: ME—Middle East; A—Africa.

**Table 5:** [24] Riatto et al., 2018.

DMF/Dmf for Different Age Groups: All Participants Were Children						
DMF-dmf/Age	5–7	8–10	11–13	6	12	5–13
DMFT	0.1	0.7	1.8	0.1	1.6	0.8
DT	0.1	0.7	1.5	0.1	1.4	0.7
MT	0	0	0.1	0	0	0
MT	0	0	0.2	0	0.2	0.1
dft	3.2	2.2	0.9	3.2	0	2.2

**Table 6:** [27] Al-Ani et al., 2020.

Detailed DMF Index: Participants Were Children and Adult								
Age group	d	m	f	dmf	D	M	F	DMF
3	2.54	0.05	0.03	2.62	-	-	-	-
6–7	4.21	0.47	0.55	5.22	0.12	0	0.02	0.13
8–11	2.50	0.53	0.57	3.60	0.42	0.02	0.26	0.70
12	0.62	0.08	0.15	0.85	1.12	0.06	0.82	2
13–17	-	-	-	-	1.93	0.23	0.72	2.87
18–34	-	-	-	-	3.72	1.46	2.24	7.43
35–44	-	-	-	-	3.13	3.22	4.21	10.55
45–64	-	-	-	-	3.64	7.63	3.64	14.92
